# Risk factors for death caused by early onset sepsis in neonates: a retrospective cohort study

**DOI:** 10.1186/s12879-023-08851-3

**Published:** 2023-11-30

**Authors:** Xinhong Chen, Huayun He, Hong Wei, Feng Chen, Ya Hu

**Affiliations:** 1 Department of Neonatology, Children’s Hospital of Chongqing Medical University, Chongqing, China; 2 National Clinical Research Center for Child Health and Disorders, Chongqing, China; 3 Ministry of Education Key Laboratory of Child Development and Disorders, Chongqing, China; 4 Chongqing Key Laboratory of Pediatrics, Chongqing, China; 5 Chongqing Higher Institution Engineering Research Center of Children’s Medical Big Data Intelligent Application, Chongqing, China

**Keywords:** Neonatal sepsis, Death, Risk, Factor, Early

## Abstract

**Objective:**

To evaluate the association between traditional laboratory findings and death, and to find risk factors for death in infants with early onset sepsis (EOS).

**Study design:**

This was a single-center, case–control, retrospective trial conducted between January 2020 and August 2021. Infants with EOS were enrolled and divided into two groups based on outcome before hospital discharge: non-survivors (Mortality group) and survivors (Survival group).

**Results:**

Out of 556 eligible neonates, there were 38 (6.8%) deaths. After univariate analysis and ROC curve analysis, there were a total of 12 values with significant differences (*p* < 0.05) between two groups, which included birth weight (BW), weight on admission, gestational age, age on admission, mode of delivery, septic shock, heart failure, respiratory failure, pulmonary hypertension, hypothermia, serum lactic acid, and aspartate aminotransferase (AST). Moreover, after multivariate analysis performed for those 12 values, the binary logistic regression analysis showed that taking death as a reference, the BW (OR = 1.00, 95% CI[1.001, 1.002], *p* < 0.001), PPHN (OR = 2.60, 95% CI[1.04, 6.52], *p* > 0.001), septic shock (OR = 6.15, 95% CI [2.52, 15.00], *p* < 0.001), heart failure (OR = 6.22, 95% CI[0.90, 43.05], *p* > 0.001), serum lactic acid (OR = 0.82, 95%CI[0.75, 0.90], *p* < 0.001), and AST (OR = 1.00, 95% CI[0.99, 1.00], *p* > 0.001) could be regarded as risk factors for death with 94.0% correct predictions.

**Conclusions:**

The factors affecting the prognosis of EOS in neonates were BW, PPHN, septic shock, heart failure, serum lactic acid, and AST. Timely correction of these modifiable risk factors for death may decrease the mortality of EOS in neonates.

## Introduction

Neonatal early onset sepsis (EOS) is a serious and fatal illness in neonatal intensive care units (NICUs) in either developed or undeveloped countries [1]. Even though the treatment for neonatal EOS has been greatly improved in recent years, it is still the third main cause of neonatal death in NICUs [2], with corresponding mortality of 11% ~ 19.0% at present, [3]. Those infants with more severe sepsis can progress to septic shock, multiple organ dysfunction syndromes rapidly and even die [4]. In view of the high mortality of neonatal EOS, increased effort is very important and needed in prevention, diagnosis, and management. Prevention is possible by assessing dead risks and providing measures that can modify or delay the development of death.

Currently, the mortality of neonatal EOS was reported mainly associated with prematurity, low birth weight, and severe infection [5, 6]. The evidence base written is somewhat limited by the small sample size or only emphasized low gestational age (GA) population [6]. To our knowledge, mortality can be reduced through effective interventions for potential risk factors that may be identified by biomarkers. However, there is little study on the biomarkers for death in neonatal EOS so far.

We assumed that some specific biomarkers can predict death may be found among traditional laboratory findings. Therefore, in this study the primary objective was to evaluate the association between traditional laboratory findings and death, and the secondary objectives were to find early risk factors for death in neonatal EOS, which may help clinicians to provide new directions for treatment to reduce the mortality of neonatal EOS.

## Materials and methods

### Definitions

1) EOS was defined as a sepsis occurring within 72 h of birth, which includes culture-positive and clinically proven EOS neonates according to the consensus of Chinese experts (2019 version) [7]: ① Culture-positive EOS neonate is a neonate who has clinical manifestations consistent with sepsis:such as lethargy, hypothermia (< 36.5℃), hyperpyrexia (> 37.5℃), poor feeding, apnea, tachypnea, cyanosis, desaturation, bradycardia (heart rate < 100 bpm for more than 5 s), tachycardia (heart rate > 160 bpm for more than 5 s)and poor perfusion), and also has a positive blood culture in the first 72 h of life② Clinically proven EOS neonates is a neonate with a negative blood culture in the first 72 h, but has manifestations consistent with sepsis and also included any one of the following conditions: nonspecific blood tests (including leukocyte count, platelet count, C-reactive protein, and procalcitonin) were ≥ 2 positive, or the cerebrospinal fluid test was purulent meningitis changes. 2) The diagnosis of septic shock was based on infants with sepsis and hypotension requiring vasopressor therapy and lactate greater than 2 mmol/L despite adequate fluid resuscitation [8]. 3) The diagnosis of bacterial meningitis was based on neonates with positive CSF culture or some cases, although with negative CSF culture, but the initial blood culture grew a pathogen, and the CSF obtained 24 to 36 h after initiation of antibiotic therapy was abnormal [9, 10]. 4) Necrotizing enterocolitis (NEC, Bell stage 2 or 3) was defined according to the classification of Bell [11]. 5) An abnormal procalcitonin (PCT) level was defined as a level with a cut-off point with time according to the age adjustment requirement as in a previous study [12]. 6) Persistent pulmonary hypertension of the newborn (PPHN) was defined according to the clinical presentation of refractory hypoxemia and echocardiographic evidence with an estimated peak systolic pulmonary-artery pressure that was higher than 35 mmHg or more than two-thirds of the systemic systolic pressure as indicated by a tricuspid regurgitant jet, a right-to-left ductus arteriosus shunt, or a right-to-left atrial-level shunt [13] 0.7) Respiratory failure was diagnosed according to the consensus of Jen-Fu Hsu et al. [14] and our institution: ① Clinical manifestations of respiratory distress, and ② requirement respiratory support using a noninvasive or invasive ventilator to maintain a target arterial blood gas: pH value > 7.25, PaO_2_ > 50 mmHg, PaCO_2_ < 55 mmHg. 9) Diagnostic criteria of heart failure was meeting all the following clinical characteristics [15]: ① shortness of breath > 60 times/min; ② tachycardia > 160 times/min; ③ heart enlargement (X-ray showed cardiothoracic ratio > 0.6 or echocardiography proven); ④ pulmonary edema, and ⑤ liver enlargement > 3 cm or galloping rhythm.

### Study design and participants

This retrospective single-center cohort study was conducted in the Department of Neonatology at Children’s Hospital of Chongqing Medical University. Infants diagnosed with neonatal EOS between January 2020 and August 2021 were included in the present study.

Human studies were reviewed and approved by the Clinical Research Ethics Committee of Chongqing Medical University (Registration number: 2022/R/181). The Ethics Committee waived the requirement for informed consent due to the anonymized nature of the data and the scientific purpose of the study.

All infants enrolled were divided into two groups based on outcome before hospital discharge: non-survivors (Mortality group) and survivors (Survival group).

### Inclusion and exclusion criteria

We tried to study the specific biomarkers for early recognition, and assessment of the risk factors of death for reducing the mortality related to neonatal EOS. Therefore, neonates were screened for inclusion: definite diagnosis of proven EOS [7] during 24–48 h after admission.

The exclusion criteria were as follows: 1) age on admission > 28 days. 2) reception of antibiotic therapy before admission to our NICU; 3) sepsis that occurred during hospitalization and considered a nosocomial infection; 4) clear genetic disease or chromosomal abnormality; 5) discharged against medical advice without sufficient antibiotic course, and 6) incomplete medical records.

The primary outcome variable was death before hospital discharge, and secondary outcomes were the first laboratory findings to our NICU among infants diagnosed with neonatal EOS.

### Data collection

Clinical data were obtained from the medical data platform of our institutional. Clinical and biological characteristics were retrospectively collected for each patient. The study data included baseline patient demographics, first laboratory data, acute comorbidities, and outcomes (survival or death).

Baseline patient demographics included meconium-stained amniotic fluid, premature rupture of membranes (PROM) > 18 h, mode of delivery, sex, gestational age, birth weight, weight on admission, and age on admission. Laboratory data including conventional inflammation markers (leukocyte count, neutrophil percentage, platelet count, C-reactive protein, and procalcitonin), arterial blood gas (pH, PCO_2_, PO_2_, HCO_3_, base deficit, serum lactic acid, and blood sugar), electrolytes (potassium, calcium, magnesium, and sodium), renal function (urea nitrogen, and serum creatinine), and hepatic function (alanine aminotransferase, aspartate aminotransferase, total protein, and albumin) were measured as early as possible after admission. Blood cultures are done on each patient at the same time. During hospitalization, only the first time of blood samples after admission of laboratory data of each patient was considered and recorded in our study. Acute comorbidities including septic shock, heart failure, respiratory failure, bacterial meningitis, necrotizing enterocolitis, persistent pulmonary hypertension, congenital heart disease, and hypothermia.

### Calculation of sample size

We assume the mortality for neonatal sepsis was about 19%, which was based on the previous study reported by Fleischmann-Struzek C et al. [3]. According to the mortality of infants suffering from neonatal sepsis of Mortality group and Survival group in the case–control study, the sample size was estimated by the software PASS 15.0 (2017. NCSS LLC, 329 North 1000 East, Kaysville, Utah, 84,037, USA. www.ncss.com.), α = 0·05, and 1-β = 0·80. The estimated sample size was 34 cases per group, and the sample size in this study was larger than the estimated required number of cases.

### Statistical analysis

All data were statistically analysed using SPSS version 26.0 (IBM, Armonk, New York) software. Categorical and continuous variables were expressed as proportions, mean ± standard deviation (M ± SD) and median (interquartile, IQR) respectively. Categorical variables were compared by the chi-square test or Fisher's exact test; Continuous variables were compared by the Mann–Whitney U-test and the t-test depending on the distributions. Moreover, the ROC curve analysis was performed in those first laboratory findings with *p* < 0.05. Prior to model development, baseline patient demographics, comorbidities (having a univariate result of *p*-value < 0.05), and the first laboratory findings (having a AUC > 0.5 and *p*-value < 0.05) were tested for multicollinearity by using variance inflation factor (VIF). A variable was removed having a VIF greater than 2.5 and VIF was repeated until all variables had a VIF less than or equal to 2.5 in Binary regression analysis. A *p*-value < 0.05 was considered statistically significant.

## Results

### Baseline patient demographics

**Fig. 1:**
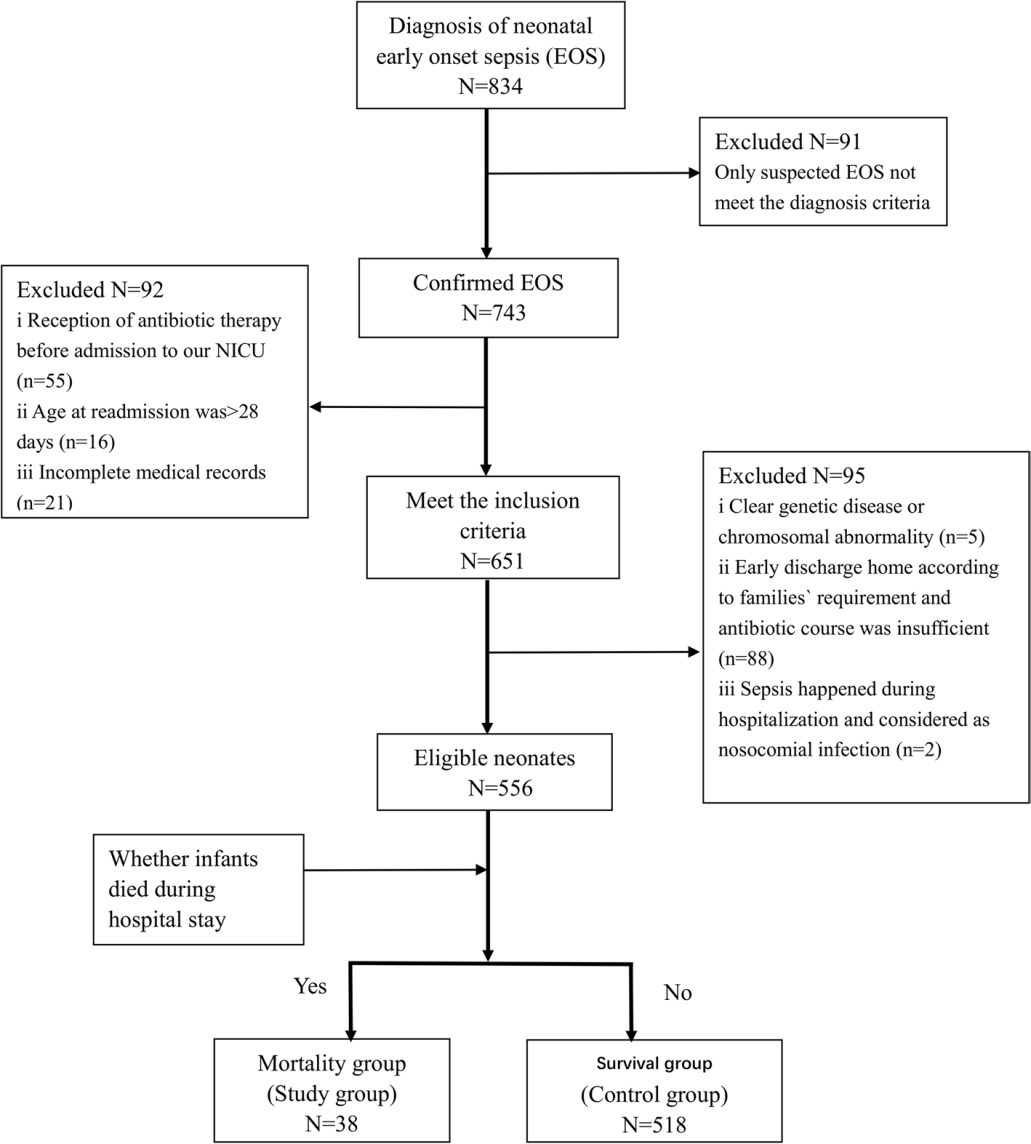
Patients selection flow chart. A total of 743 patients diagnosed proven NS were admitted to our NICU. 187 neonates who met the exclusion criteria were excluded. Thus, 556 neonates were analyzed in the present study. There were 38 (6.8%) non-survivors who were divided into the Mortality group, and the other 518 (93.2%) survivors were divided into the Survival group

From January 2020 to August 2021, a total of 743 patients diagnosed proven neonatal EOS were admitted to our NICU. 187 neonates who met the exclusion criteria were excluded. Thus, 556 neonates were analyzed in the present study. There were 38 (6.8%) non-survivors who were divided into the Mortality group, and the other 518 (93.2%) survivors were divided into the Survival group. The inclusion process is shown in Fig. 1.

The Mortality group had significantly lower BW, lower weight on admission, smaller GA, and younger age on admission than the Survival group [1819.5 ± 932.3 g vs 2895.9 ± 847.2 g, *p* < 0.001; 1828.9 ± 1000.6 g vs 2867.2 ± 872.2 g, *p* < 0.001; 31.8 (26.7,40.3) weeks vs 38.6 (35.0,39.9)weeks, *p* < 0.001; 16.5 (11.3,20.0) hours vs 25.5 (11.8,64.5) hours, *p* < 0.001, respectively]. Moreover, cesarean section occurred more frequently in the Mortality group than the Survival group (76.3% vs 54.6%). In contrast, there were no differences in sex, meconium-stained amniotic fluid, or PROM, as shown in Table 1.

**Table 1:** Clinical characteristics in neonates wssith sepsis

Variables	Total (*N* = 556)	Mortality group (*N* = 38)	Survival group (*N* = 518)	*p*-value
**Demographics of neonates and their mothers**				
**Neonatal factors**				
Male sex, n (%)	347 (62.4)	24 (63.2)	323 ( 62.4)	0.921
Birth Weight (g) ^a d^	2821.8 ± 895.0	1819.5 ± 932.3	2895.9 ± 847.2	< 0.001
Weight at admission (g) ^a^	2796.3 ± 918.8	1828.9 ± 1000.6	2867.2 ± 872.2	< 0.001
Birth GA (weeks) ^**b**^	38.4 (34.5,39.9)	31.8 (26.7,40.3)	38.6 (35.0,39.9)	< 0.001
Age at admission (hours) ^b^	24.2 (16.2,61.9)	16.5 (11.3,20.0)	25.5 (11.8,64.5)	< 0.001
Age of onset (hours)^b^	1.0 (1.0,24.0)	1.0(1.0,1.0)	1.0 (1.0,24.0)	< 0.001
Premature infants, n (%)	203 (36.5)	30 (78.9)	173 (33.4)	< 0.001
Multiple births, n (%)	68 (12.2)	12 (31.6)	56 (10.8)	0.001^c^
**Maternal factors**				
Cesarean section, n (%)	312 (56.1)	29 (76.3)	283 (54.6)	0.009
Meconium stained amniotic fluid, n (%)^e^	114 (20.5)	11 (28.9)	103 (19.9)	0.408
PROM > 18 h, n (%)	45 (8.1)	2 (5.3)	43 (8.3)	0.599
Intrauterine distress, n (%) ^f^	80 (14.4)	13 (34.2)	67 (12.9)	0.001
Placental abruption, n (%) ^g^	45 (8.1)	2 (5.3)	43 (8.3)	0.599
Cesarean section, n (%)	312 (56.1)	29 (76.3)	283 (54.6)	0.009
**Acute comorbidities with sepsis**				
Septic shock, n(%)	48 (8.6)	18 (47.4)	30 (5.8)	< 0.001^c^
Heart failure, n(%)	6 (1.1)	4 (10.5)	2 (0.4)	< 0.001^c^
Respiratory failure, n(%)	275 (49.5)	34 (89.5)	241 (46.5)	< 0.001
Bacterial meningitis, n(%)	15 (2.7)	2 (5.3)	13 (2.5)	0.273^c^
NEC, n(%)	104 (18.7)	12 (31.6)	92 (17.8)	0.035
PPHN, n(%)	91 (16.4)	21 (55.3)	70 (13.5)	< 0.001
Congenital heart disease, n(%)	294 (52.9)	21 (55.3)	273 (52.7)	0.402
Hypothermia, n (%)	30 (5.4)	11 (28.9)	19 (3.7)	< 0.001^c^

*GA* gestational age, *RR; PROM* premature rupture of membranes, *NEC* Necrotizing enterocolitis, *PPHN* persistent pulmonary hypertension

^a^Mean and standard deviation

^b^Median and interquartile range

^c^Fisher’s exact test

^d^4 (0.7%) infants had not obtained their birth weight and were excluded from this variable

^e^68 (12.2) infants did not know the nature of amniotic fluid and were excluded from this variable

^f^94 (16.9) infants did not know they whether had intrauterine distress and were excluded from this variable

^g^7 (1.3) infants did not know their mother had a placental abruption and were excluded from this variable

### Acute comorbidities

Septic shock, heart failure, respiratory failure, NEC, PPHN and hypothermia all occurred more frequently in the Mortality group than the Survival group (47.4% vs 5.8%, *p* < 0.001; 10.5% vs 0.4%, *p* < 0.001; 89.5% vs 46.5%, *p* < 0.001; 31.6% vs 17.8%, *p* = 0.035; 55.3% vs 13.5%, *p* < 0.001; 28.9% vs 3.7%, *p* < 0.001, respectively). On the other hand, there were no differences in the morbidity of bacterial meningitis, congenital heart disease, or ABO hemolytic disease between the two groups, as shown in Table 1.

### First laboratory findings

Compared with the Survival group, the Mortality group had significantly lower level / percentage of those variables: platelet count (150.5 ± 75.6 *10^9^/L vs 255.7 ± 105.1 *10^9^/L, *p* < 0.001), neutrophil percentage (59.1 ± 14.4% vs 64.6 ± 16.5%,*p* = 0.045), CRP > 8 mg/L (10.5% vs 34.4%, *p* = 0.009), abnormal PCT ( 23.7% vs 34.7%,*p* < 0.001), pH [ 7.32 (7.21,7.40) vs 7.40 (7.32,7.46), *p* = 0.002], HCO_3_ [20.4 (16.2,22.9) mmol/L vs 22.6 (20.9,24.4) mmol/L, *p* = 0.006], base deficit [-5.1 (-9.4,-3.2) mmol/L vs -2.9 (-5.2,-0.6) mmol/L, *p* = 0.002], Na [ 135.9(132.7,138.2) mmol/L vs 137(135.1,139.0) mmol/L, *p* = 0.045], Ca [2.1 (1.9,2.3) mmol/L vs 2.3 (2.1,2.4) mmol/L, *p* = 0.019], total protein (44.3 ± 8.7 g/L vs 53.1 ± 8.0 g/L, *p* < 0.001), and albumin ( 25.9 ± 6.1 g/L vs 31.9 ± 5.3 g/L, *p* < 0.001). Meanwhile, the Mortality group had significantly higher level of blood lactic acid [4.5 (2.8,9.1) mmol/L vs 2.6 (1.8,3.7) mmol/L, *p* < 0.001] and AST [ 64.4(45.9,107.9).

U/L vs 51.0 (36.2,73.3) U/L, *p* = 0.028]. On the contrary, other laboratory findings (including leukocyte count, PCO_2_, PO_2_, blood sugar, K, Mg, ALT, urea nitrogen, and serum creatinine) were not significantly different between the two groups (all *p* > 0.05) (Table 2).

**Table 2:** The biomarkers in neonates with sepsis

Variables	Total (*N* = 556)	Mortality group (*N* = 38)	Survival group (*N* = 518)	*p*-value
**Traditional markers of inflammation**				
Platelet count ^a^	248.5 ± 106.6	150.5 ± 75.6	255.7 ± 105.1	< 0.001
Neutrophil percentage(%) ^a^	64.2 ± 16.4	59.1 ± 14.4	64.6 ± 16.5	0.045
Leukocyte count (*10^9^/L) ^b^	14.2 (9.3,20.8)	14.7(6.9,30.2)	14.2(9.3,20.8)	0.867
CRP > 8 mg/L, n(%) ^c^	182 (32.7)	4 (10.5)	178 (34.4)	0.009
Abnormal PCT (ng/L),n(%) ^g^	189 (34.0)	9 (23.7)	180 (34.7)	< 0.001
**Blood gas**^**b**^				
pH ^d^	7.39 (7.31,7.46)	7.32 (7.21,7.40)	7.40 (7.32,7.46)	0.002
PCO2 (mmol/L) ^d^	35.0 (28.0,43.0)	37.0 (28.0,47.0)	35.0 (28.0,42.8)	0.613
PO2 (mmol/L) ^d^	76.0 (57.0,101.0)	95.0 (55.5,139.0)	75.0 (58.0,99.0)	0.259
HCO3 (mmol/L) ^e^	22.5 (20.7,24.3)	20.4 (16.2,22.9)	22.6 (20.9,24.4)	0.006
Base deficit (mmol/L) ^e^	-3.0 (-5.3,-0.8)	-5.1 (-9.4,-3.2)	-2.9 (-5.2,-0.6)	0.002
Blood sugar (mmol/L) ^d^	4.2 (3.4,5.3)	4.3 (3.3,5.9)	4.2 (3.4,5.3)	0.953
Blood lactic acid (mmol/L) ^e^	2.70 (1.9,3.9)	4.5 (2.8,9.1)	2.6 (1.8,3.7)	< 0.001
**Other biochemical indicators**				
Na (mmol/L) ^b^	136.9(135.0,139.0)	135.9(132.7,138.2)	137(135.1,139.0)	0.045
K (mmol/L)^**b**^	4.7 (4.4,5.1)	4.5 (3.9,5.6)	4.7 (4.4,5.1)	0.278
Ca (mmol/L)^**b**^	2.3 (2.1,2.4)	2.1 (1.9,2.3)	2.3 (2.1,2.4)	0.019
Mg (mmol/L)^**b**^	0.8 (0.7,0.9)	0.9 (0.7,1.2)	0.8 (0.7,0.9)	0.197
ALT (U/L^b,f^	19.7 (15.1,25.3)	18.4 (14.3,28.4)	19.8 (15.1,25.3)	0.622
AST (U/L)^b,f^	51.5 (36.5,76.0)	64.4 (45.9,107.9)	51.0 (36.2,73.3)	0.028
Total protein (g/L) ^a,f^	52.5 ± 8.4	44.3 ± 8.7	53.1 ± 8.0	< 0.001
Albumin (g/L)^a,f^	31.5 ± 5.6	25.9 ± 6.1	31.9 ± 5.3	< 0.001
Blood urea nitrogen (mg/dL)^**b**^	3.7 (2.9,4.8)	4.1 (3.4,5.2)	3.7 (2.9,4.8)	0.239
Blood serum creatinine (mg/dL)^a^	60.4 ± 30.9	63.3 ± 20.5	60.2 ± 31.5	0.554

*CONS* coagulase-negative staphylococcus*, K* potassium concentration, *Ca* calcium concentration, *Mg* magnesium concentration, *Na* sodium concentration, *ALT* alanine aminotransferase, *AST* aspartate aminotransferase

^a^Mean and standard deviation

^b^Median and interquartile range

^c^2 (0.4%) infants had not checked their CRP and were excluded from this variable

^d^19 (3.4%) infants had not obtained their PH, PO2, PCO2, or Blood sugar and were excluded from these variables

^e^21 (3.8%) infants had not obtained their HCO3, Base deficit, or Blood lactic acid and were excluded from these variables

^f^1 (0.2%) infant had not obtained his AST, and ALT and was excluded from these variables

^g^13 (2.3%) infants had not checked their PCT and were excluded from this variable

^h^Fisher’s exact test

**Fig. 2:**
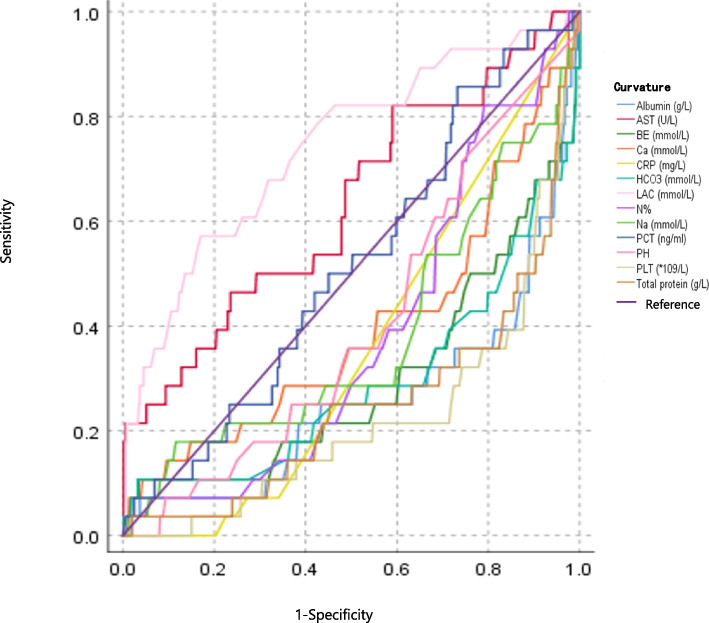
ROC Curve of prognostic values of first laboratory findings for death in neonates with sepsis. ROC curve analysis was performed to further understand the relationship between those variables (having a *p* < 0.05) and death

ROC curve analysis was performed to further understand the relationship between those variables (having a *p* < 0.05) and death. Only serum lactic acid (having an AUC of 0.742 and at the cut-off value of 4.4 mmol/L) and AST (having an AUC of 0.632 and at the cut-off value of 45.6 U/L) were found obvious correlation with death, which had an AUC > 0.5 and *p* < 0.05. However, other values (including platelet count, neutrophil percentage, CRP > 8 mg/L, abnormal PCT, pH, HCO3, base deficit, Na, Ca, total protein, and albumin) did not prove this correlation, because they all can't be satisfied with an AUC > 0.5 and *p* < 0.05 at the same time (Table 3, Fig. 2).

**Table 3:** Prognostic values of first laboratory findings for death

Variables	AUC	Cutoff	Sensitivity (%)	Specificity (%)	*p*- value	95%CI
PLT (*10^9^/L)	0.222	20.0	100.0	0.4	< 0.001	0.130 ~ 0.315
N %	0.381	86.5	14.3	96.4	0.014	0.286 ~ 0.476
CRP (mg/L)	0.361	11.5	7.1	72.8	0.001	0.276 ~ 0.445
PCT (ng/ml)	0.513	0.3	85.7	26.8	0.806	0.409 ~ 0.617
pH	0.406	7.5	7.1	90.7	0.066	0.306 ~ 0.506
HCO_3_ (mmol/L)	0.293	27.1	10.7	96.8	0.001	0.175 ~ 0.411
BE (mmol/L)	0.297	2.8	10.7	96.6	0.001	0.182 ~ 0.413
Blood Lac (mmol/L)	0.742	4.4	57.1	83.0	< 0.001	0.638 ~ 0.845
Na (mmol/L)	0.382	140.8	17.9	88.4	0.051	0.264 ~ 0.501
Ca (mmol/L)	0.397	2.6	10.7	95.7	0.095	0.276 ~ 0.518
AST (U/L)	0.632	45.6	82.1	41.0	0.024	0.517 ~ 0.746
Total protein (g/L)	0.25	68.6	3.6	98.2	< 0.001	0.147 ~ 0.352
Albumin (g/L)	0.26	40.4	7.1	96.3	< 0.001	0.149 ~ 0.370

*PLT* Platelet Platelet count, *N* Neutrophil percentage, *PCT* Procalcitonin, *CRP* C-reactive protein, *Lac* Lactic acid, *AUC* Area Under Curve, *AST* Aspartate aminotransferase

### Distribution of pathogens in blood cultures

Blood culture tests were performed in all infants, and positive cultures were obtained in 47 (8.5%) infants. There was no significant difference in positive rate of blood culture and multiple drug-resistant bacteria between the Mortality group and Survival group (10.5% vs 8.3%, *p* = 0.551; 25.0% vs 30.2%, *p* = 1.000, respectively). Gram-negative bacilli were the main pathogens in positive blood cultures, followed by CoNS, as shown in Table 4.

**Table 4:** Blood Culture in neonates with sepsis

Variables	Total (*N* = 556)	Mortality group (*N* = 38)	Survival group (*N* = 518)	*p*-value
Culture positive	47 (8.5)	4 (10.5)	43 (8.3)	0.551
Multiple drug-resistant bacteria	14 (29.8)	1 (25.0)	13 (30.2)	1.000
Distribution of pathogens)				
Gram-negative bacteria	19 (3.4)	3 (7.9)	16 (3.1)	1.000
CoNS*	15 (2.7)	1 (2.6)	14 (2.7)	
Listeria monocytogenes	3 (0.5)	0 (0.0)	3 (0.6)	
Others	10 (1.8)	0 (0.0)	10 (1.9)	

^*^CONS coagulase-negative staphylococcus

### Logistic regression analysis of risk factors associated with death

First, important covariates were derived from the univariate analysis and ROC curve analysis, which included demographic characteristics, and comorbidities (having a univariate result of *p*-value < 0.05) and the first traditional laboratory findings (having a AUC > 0.5 and *p*-value < 0.05). Hence, a total of 13 values, including BW, weight on admission, GA, age on admission, mode of delivery, septic shock, heart failure, respiratory failure, pulmonary hypertension, hypothermia, serum lactic acid, and AST were further analysed in the multivariable analysis by using variance inflation factor (VIF). After variance inflation factor analysis, two values (including weight on admission, and GA with a VIF > 2.5) were excluded. Therefore, only 11 values left were included in the final multivariate logistic regression.

Then, taking death as a reference, the binary logistic regression analysis showed that BW (OR = 1.001, 95% CI[ 1.001, 1.002], *p* < 0.001), PPHN (OR = 2.604, 95% CI[1.041, 6.515], *p* = 0.041), septic shock (OR = 6.151, 95% CI [2.523, 14.997], *p* < 0.001), heart failure (OR = 6.217, 95% CI [0.898, 43.049], *p* = 0.064), serum lactic acid (OR = 0.818, 95% CI [0.745, 0.898], *p* < 0.001), and AST (OR = 0.999, 95% CI [0.998, 1.000], *p* = 0.049) were independent risk factors for death. The best logistic regression model was administered: Logistic (Death) = -3.527 + 0.001 X1(BW) + 0.957 X2 (PPHN) + 1.817 X3 (septic shock) + 1.827 X4 (heart failure)-0.201 X5 (serum lactic acid)-0.001 X6 (AST) (X^2^ = 113.096, *p* < 0.001), with 94.0% correct predictions, as shown in Table 5.

**Table 5:** Multivariate analysis of predictors for death in neonates with sepsis

Variables	β	S.E	Wald	*p*-value	OR	95% CI
BW (X1)	0.001	0.000	22.922	< 0.001	1.001	1.001 ~ 1.002
PPHN ( X2)	0.957	0.468	4.186	0.041	2.604	1.041 ~ 6.515
Septic shock ( X3)	1.817	0.455	15.958	< 0.001	6.151	2.523 ~ 14.997
Heart failure( X4)	1.827	0.987	3.425	0.064	6.217	0.898 ~ 43.049
LAC ( X5)	-0.201	0.047	17.963	< 0.001	0.818	0.745 ~ .898
AST (× 6)	-0.001	0.001	3.891	0.049	0.999	0.998 ~ 1.000

*LAC* serum lactic acid, *PPHN* persistent pulmonary hypertension of the newborn, *AST* Aspartate aminotransferase

## Discussion

The results of this study showed that the mortality of neonatal early onset sepsis was 6.8%, a little lower than the previous studies [2, 3] due to improvement of medical level in recent years. However, the mortality of neonatal sepsis still remained high. Thus, investigating the specific biomarkers for risk factors in septic infants may be helpful to decrease the mortality of neonatal EOS.

In our study, main pathogens were gram-negative bacilli and CoNS, which is similar with Wang J et al. [16] in China, while Group B hemolytic streptococcus (GBS) and Listeria are more common in Europe and America [17].

Our study found that BW, PPHN, septic shock, heart failure, serum lactic acid, AST were independent risk factors for death in neonatal EOS. The above indicators are easy to obtain clinically. It is also simple to facilitate the early identification and risk stratification of high-risk neonates, so it has strong clinical practicability.

Infants with low birth weight are more likely to die when they suffer from severe infection due to their immature development [1–3]. The similar finding was proven in the study, which showed deaths having much lower BW than survivors. Therefore, for infants with a birth weight fewer than 1819.5 g, if sepsis occurs, vigilance must be paid to the possibility of death due to deterioration of the disease.

Another independent risk factor for death in neonatal EOS in this study was PPHN. PPHN in neonates is one of the most severe neonatal diseases [18]. When pulmonary hypertension is not corrected, it will lead to continuous hypoxia and hypoperfusion state, then worsen systemic inflammation as a vicious cycle [19]. Thus, aggressive therapy for PPHN, such as inhaled nitric oxide, might reduce the occurrence of death in septic infants.

Septic shock was the third independent risk factor for death. In shock states, blood flow to the brain and heart is maintained owing to the redistribution of blood away from peripheral organs, enhances bacterial translocation, and leads to multiple organ dysfunction [20]. Thus, in neonatal EOS with clinical suspicion of shock, signs of shock must be actively examined because timely treatment of this complication is critical to prognosis.

An association between heart failure and death has been proven by previous study [15]. Our findings have indicated that infants with heart failure having 6.217 times more likely to die than those without it. Our results emphasize the importance of heart function in neonatal EOS, which will help clinicians better understand infant outcomes.

Meanwhile, serum lactic acid was confirmed to be associated with death, which agrees with the findings of Chaudhry S et al. [21]. Andersen LW et al. [22] indicated that serum lactic acid is a universally accepted, clinically helpful indicator of tissue hypoperfusion. Hypoperfusion pressure prevents nutrient and oxygen supply to peripheral tissue, which may further impede the healing process and damage the integrity of multiple organs. In addition, Rhodes et al. [23] found that patients with persistent elevation of serum lactic acid had a significantly increased risk of refractory shock and multiple organ dysfunction. Furthermore, serum lactic acid at the cut-off value of 4.4 mmol/L had an AUC of 0.742 in predicting death risk, with a sensitivity of 57.1% and a specificity of 83.0% on the first blood sample. Hence, elevated serum lactic acid > 4.4 mmol/l indicates a high risk for death of NS.

Secondly, AST was also associated with death in neonatal EOS. Oswari H et al. [24] concluded that AST could be used to predict the poor prognosis of neonatal sepsis-related cholestasis, such as severe sepsis, septic shock, and even death. The increase in AST most likely reflects a rapid reduction in in liver function [25], especially observed in neonates once decompensation occurs.

The main strength of our study is that it may be the largest data analyzed for the association between traditional laboratory findings and death, and maybe the first retrospective cohort study focused on the early risk factors for death in neonatal early onset sepsis. In this study, we found potential independent risk factors associated with death, such as PPHN, septic shock, and serum lactic acid etc., which may help clinicians timely act intervention to reduce neonatal mortality of EOS [20].

We acknowledge some limitations in our study. First, we only included infants treated with antibiotics after admission to our NICU, and further studies are needed to determine whether infants treated before admission have similar clinical characteristics. Second, our study did not include the content of treatment, and relevant research can be carried out in the future. Third, the gestation age in our study is basically 38.4 weeks (most 34.5–39.9 weeks) and there only 14 cases (2.5%) with gestation age < 28 weeks, which is a limitation to our study relative to previously reported studies focused on more small gestational age in different regions. The difference in mortality may be mainly related to the fact that the gestational age of the newborns included in our study was not as small as previously reported, so our average NICU mortality was lower, which may impact the generalizability of our findings.

## Conclusion

In conclusion, our findings explored the possible risk factors for the death of neonatal EOS and extended the current understanding with respect to relevant clinical outcomes. In our study, the factors affecting the prognosis of EOS were BW, PPHN, septic shock, heart failure, serum lactic acid, and AST. Timely correction of these modifiable risk factors for death could decrease the mortality of EOS, and our data may help to provide the rationale for a strategy of prevention and optimal interventions in the future.

## Data Availability

The original contributions presented in the study are included in the article, further inquiries can be directed to the corresponding author.

## Abbreviations

ALT: Alanine aminotransferase
AST: Aspartate aminotransferase
AUC: Area under the curve
Ca: Calcium concentration
CoNS: Coagulase-negative staphylococci
CRP: C-reactive protein
GA: Gestational age
K: Potassium concentration
LAC: Serum lactic acid
Mg: Magnesium concentration
EOS: Early onset sepsis
NICUs: Neonatal intensive care units
NEC: Necrotizing enterocolitis
Na: Sodium concentration
PCT: Procalcitonin
PPHN: Persistent pulmonary hypertension of the newborn
PROM: Premature rupture of membranes
PLT: Platelet count
ROC: Receiver operating characteristic
